# Individuals prefer to harm their own group rather than help an opposing group

**DOI:** 10.1073/pnas.2215633119

**Published:** 2022-11-29

**Authors:** Rachel Gershon, Ariel Fridman

**Affiliations:** ^a^Rady School of Management, UC San Diego, San Diego, CA 92093

**Keywords:** intergroup conflict, identity, norms, decision-making, polarization

## Abstract

Group-based conflict enacts a severe toll on society, yet the psychological factors governing behavior in group conflicts remain unclear. Past work finds that group members seek to maximize relative differences between their in-group and out-group (“in-group favoritism”) and are driven by a desire to benefit in-groups rather than harm out-groups (the “in-group love” hypothesis). This prior research studies how decision-makers approach trade-offs between two net-positive outcomes for their in-group. However, in the real world, group members often face trade-offs between net-negative options, entailing either losses to their group or gains for the opposition. Anecdotally, under such conditions, individuals may avoid supporting their opponents even if this harms their own group, seemingly inconsistent with “in-group love” or a harm minimizing strategy. Yet, to the best of our knowledge, these circumstances have not been investigated. In six pre-registered studies, we find consistent evidence that individuals prefer to harm their own group rather than provide even minimal support to an opposing group across polarized issues (abortion access, political party, gun rights). Strikingly, in an incentive-compatible experiment, individuals preferred to subtract more than three times as much from their own group rather than support an opposing group, despite believing that their in-group is more effective with funds. We find that identity concerns drive preferences in group decision-making, and individuals believe that supporting an opposing group is less value-compatible than harming their own group. Our results hold valuable insights for the psychology of decision-making in intergroup conflict as well as potential interventions for conflict resolution.

## Significance

Understanding the principles guiding decisions in intergroup conflicts is essential to recognizing the psychological barriers to compromise and cooperation. We introduce a novel paradigm for studying group decision-making, demonstrating that individuals are so averse to supporting opposing groups that they prefer equivalent or greater harm to their own group instead. While previous models of group decision-making claim that group members are driven by a desire to benefit their in-group (“in-group love”) rather than harm their out-group, our results cannot be explained by in-group love or by a harm minimizing strategy. Instead, we propose that identity concerns drive this behavior. Our theorizing speaks to research in psychology, political theory, and negotiations by examining how group members navigate trade-offs among competing priorities.

Group conflicts are a pervasive feature of society. Yet, despite the extensive literature on the topic, a unified understanding of the psychology underlying decision-making in group conflicts remains elusive. Prior work has documented two broad principles governing group-based decision-making. First, group members exhibit in-group favoritism (1, 2). That is, individuals prefer to create a favorable comparison between their in-group and the out-group, even leading to choices that prioritize relative gains compared to the out-group over greater absolute gains for their in-group (see Social Identity Theory; refs. 3 and 4). Second, past work theorizes that group members are driven by a cooperative motive to help the in-group (“in-group love”) rather than an aggressive motive to hurt the out-group (“out-group hate”; 5, 6). Critically, these principles are derived from studies in which participants chose between outcomes that are all ultimately favorable to the in-group. However, real-world decision-making often entails making choices where harm is unavoidable (7, 8). Groups may have to choose between in-group losses and out-group gains, a circumstance that has not been previously studied and reveals that individuals’ decisions cannot be explained by existing theories.

Consider the example of Montgomery, Alabama, where only White residents were allowed to use the publicly funded Oak Park Pool until, in 1959, a federal court deemed the segregated pool unconstitutional. The White town council then faced two options that they considered unfavorable: give Black families access to the pool or close one of the town’s favorite gathering spots. Previous work on in-group favoritism and the dominant role of “in-group love” would predict that white Montgomery residents would avoid harming their own group even at the cost of extending pool use to Black citizens. Yet famously, the white citizens of Montgomery closed the pool. Other public resources, such as parks and zoos, were closed to all across the country to defy similar rulings (9). The Oak Park Pool is an exemplar of the fact that, anecdotally, when faced with two counter-attitudinal choices—aid the out-group or harm the in-group —group members may avoid showing support for the out-group, even at the apparent expense of their own side (10–13). However, there has never been a rigorous investigation of how individuals navigate unfavorable choices in intergroup conflicts and whether there is a broad preference to harm one’s own group rather than support the opposition.

Here, we develop a novel paradigm in which individuals must either deduct funds from their in-group or add funds to an opposing group to examine how group members make trade-offs in lose-lose* intergroup conflicts. Our experiments were conducted across multiple countries (United States and United Kingdom), several polarized issues (abortion access, political party, and gun control), and various experimental measures (financial donations and incentive-compatible multiple price lists). Taken together, our results offer the first unambiguous evidence that individuals are so averse to showing support for an opposing out-group that they even prefer to do greater harm to their own group instead. Our finding was symmetrically exhibited by individuals on both sides of each issue we studied, and even among participants who identified only weakly with their side. However, the degree to which a participant identified with their side of the focal issue does play a moderating role—those with stronger attitudes in favor of their side (e.g., more strongly pro-choice) were more likely to choose to harm their own side (and willing to cause greater overall harm to their side) rather than help the opposing side.

Our results reveal the central role of identity in decision-making in polarized contexts. Identity often plays an important role in decision-making (14), such as when and to whom we offer support (15, 16). We propose that individuals aim to protect their group-based identity when facing intergroup conflict and therefore behave in ways that best express their values, especially those that are central to their identity (17). Previous work finds that individuals prefer expressions of support (e.g., “I support Democrats”) to expressions of opposition (e.g., “I oppose Republicans”), because support is considered more “value-expressive” (18, 19). We would therefore expect group members to choose actions of support over actions of opposition when both options convey their values (i.e., choosing to help their own side rather than harm their opponent). However, what would individuals choose when the options are unfavorable, inconsistent with their values (i.e., a lose-lose choice between harming their own group or helping their opponent)? In such situations, since both options express values that are counter-attitudinal, our Identity-Support model suggests that individuals will choose the least value-expressive option, thereby best protecting their identity. Our results support these predictions—individuals believe that helping an opposing group is more harmful to their identity than inflicting equivalent harm to their in-group, even when this leads to a worse relative standing for their in-group. Critically, we find that by ameliorating identity concerns through shifting perceived in-group norms (20–24), individuals become more likely to support the opposing group, providing a practical way to achieve more constructive outcomes in group conflict.

While there are numerous existing models of group decision-making, the Identity-Support model can uniquely explain individual behavior for both favorable (win-win) and unfavorable (lose-lose) choices with opposing groups. We address other frameworks below in Table 1. First, it is possible that individuals view these decisions as a zero-sum trade-off (25, 26), wherein a gain for one group is perceived as an equivalent loss for the other group. In this case, group members should be indifferent between the two options. It is also possible that group members consider which side is more effective at using funds to pursue their mission and then choose based on harm minimization for their cause. Across our studies, we find that group members typically believe their own group is more effective with funds, so those motivated by harm minimization should prefer to add to their own (more effective) side in win-win scenarios and avoid subtracting from their own side in lose-lose scenarios.

**Table 1.** Group decision-making theories. The ✓ indicates that the theory would predict a preference for the given choice, whereas 50% indicates an indifference between the choices

	Win-Win		Lose-Lose	
	Add $1 to in-group	Subtract $1 from opposing group	Add $1 to opposing group	Subtract $1 from in-group
**Identity-support model**	**✓**			**✓**
Zero-sum beliefs	50%	50%	50%	50%
Harm minimization (based on effectiveness considerations)	✓		✓	
Maximize in-group payoff	✓		✓	
Maximize relative payoff for in-group	50%	50%	50%	50%
Minimize payoff difference	50%	50%	50%	50%
Maximize joint payoff, in favor of in-group	✓		50%	50%
Minimize opposing group payoff		✓		✓

The social value orientation literature (e.g., ref. 27), which arbitrarily assigns in-groups and out-groups (i.e., a minimal group paradigm) to study group member decision-making, describes additional motives that may also guide decision-making for real-world opposing groups. For example, individuals may simply prefer allocations that maximize the payoff for the in-group (analogous to being motivated by in-group love (5, 6)), maximize the relative difference in payoff between their in-group and opposing group (consistent with findings of in-group favoritism (1, 2)), or, as demonstrated in Bornstein et al., (1983), either minimize the difference in payoffs to each side or maximize joint profit in favor of the in-group. Additionally, recent work on negative partisanship and increased out-group animosity (28–30) suggests that individuals may be primarily motivated to minimize the payoff to the opposing side. Table 1 summarizes how individuals guided by each of these motives would choose when faced with win-win and lose-lose scenarios. Notably, the Identity-Support model is the only model that can explain both findings across scenarios.

Finally, previous work on prospect theory (31, 32), finding that people experience losses more strongly than equivalent gains, also does not make clear predictions in this context. For example, when faced with a lose-lose scenario, while participants technically choose between a loss or gain (of funding), they may encode both adding to the opposing group and subtracting from the in-group as losses for their side. That is, any gains for the opposing group can feel like losses for the in-group and vice versa. However, if this is not the case and individuals are indeed more affected by losses to their side than gains to the opposing side, then our findings would be inconsistent with predictions based on prospect theory as well.

## Open Science

All study designs and analyses were pre-registered, and all data, analysis code, research materials, and pre-registrations are available at https://osf.io/gzxke/. Data were analyzed using R, version 4.1.2, and the package ggplot2, version 3.3.5 (33). For all studies, we reported all manipulations and measures and recruited a minimum of 100 participants per condition. All sample sizes and exclusion criteria were determined in advance.

## Results

### Study 1: Individuals Prefer to Harm Their Own Group Rather Than Support the Opposition.

In Study 1, we develop a novel paradigm to investigate how individuals behave in group conflicts in which they must choose between supporting an opposing group or harming their own group. In our paradigm, we asked participants to indicate their position on several polarized issues in two sub-studies conducted with participants from the United States and United Kingdom. Given the similarities in their designs and hypotheses, we report these studies together, noting only where they differ. In the US study (Study 1A; N = 797, matched to US census data on age, sex, and ethnicity), issues included abortion access, gun control, and political party affiliation (Democratic or Republican Party). In the UK study (Study 1B; N = 393, matched to UK census data on age, sex, and ethnicity), participants were asked about political party affiliation (Labor or Conservative Party). After indicating their attitude toward the relevant issues (on a six-point Likert scale^†^, which we subdivide into weak, medium, and strong attitudes; see *Methods* section), participants learned that real donations would be made to organizations supporting each side of the partisan divide (e.g., both a pro-choice and pro-life organization). Participants were then asked how they would choose to alter the donation (in Study 1A, they rated each of the three issues separately, randomly ordered) and were informed that for ten randomly chosen participants, their choices would actually be implemented. Participants were randomly assigned to one of two between-subjects experimental conditions: (a) a win-win condition or (b) a lose-lose condition. In the win-win condition, which serves as an experimental control to conceptually replicate prior findings (5, 6), both options altered the donation in ways that were favorable given the participant’s stated attitude: either add $1 to the donation going to the organization on their side or subtract $1 from the donation going to the organization on the opposing side. In the lose-lose condition, both options altered the donation in ways that were unfavorable given the participant’s stated attitude: either add $1 to the donation going to the organization on the opposing side or subtract $1 from the donation going to the organization on their side. Finally, participants reported which side of each cause was more effective at using funds to pursue their mission. Specifically, they were asked “Do you believe that [Pro-life/Pro-gun/Republican/Conservative] or [Pro-choice/Anti-gun/Democratic/Labor] organizations are more effective at pursuing their mission? In other words, which one is able to do more with each dollar they receive?”

**Fig. 1.**
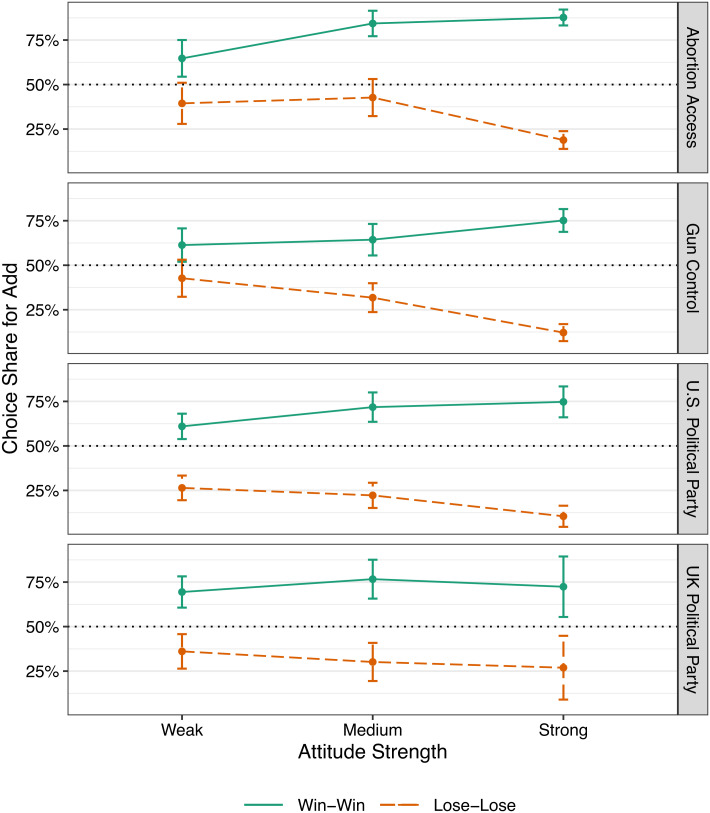
Choice share in each condition for all four issues by attitude strength in Studies 1A (N = 797) and 1B (N = 393). The vertical axis shows the proportion of participants choosing to add funds (in the lose-lose condition: add funds to opposing group vs. subtract from their in-group; in the win-win condition: add funds to the in-group vs. subtract from the opposing group). Error bars represent 95% confidence intervals.

In the win-win condition, our results conceptually replicate and extend prior studies. We find that participants are more likely to choose to support their side by adding $1 to the organization on their side of each cause (72.5%) rather than harm the opposition by subtracting $1, t(594) = 13.87, *P* < 0.001, and 95% CI = [69.7%, 75.2%]^‡^ (see Fig. 1). Thus, these results reproduce the findings of the in-group love model in the context of a win-win choice in our sample, extending previous results to natural groups (as opposed to minimal group paradigms).

The results of our lose-lose condition, however, contradict the predictions of the in-group love model. While in-group love predicts that individuals will support their opposition to avoid harming their in-group, participants in the lose-lose condition predominantly chose not to help the opposition (helping the opposing group was chosen by only 25.8% of participants), preferring instead to harm their own group almost three-quarters of the time, t(594) = −13.85, *P* < 0.001, and 95% CI = [23.0%, 28.7%] (see Fig. 1).

The preference to harm the in-group rather than support the opposition is robust and consistent across a variety of subsamples. First, we find similar effects for both the nationally representative sample in the United States (24.6%; t(398) = −12.92, *P* < 0.001, and 95% CI = [21.6%, 27.9%]) and the nationally representative sample in the United Kingdom (32.7%; t(195) = −4.74, *P* < 0.001, and 95% CI = [26.4%, 39.5%]). Second, the same pattern holds across every issue we tested (abortion: (27.8%; t(398) = −8.52, *P* < 0.001, and 95% CI = [23.6%, 32.4%]); gun control: (25.3%; t(398) = −9.39, *P* < 0.001, and 95% CI = [21.3%, 29.8%]); party support: (24.7%; t(594) = −11.71, *P* < 0.001, and 95% CI = [21.4%, 28.3%]). Finally, the preference to harm the in-group rather than support the opposition is present on both sides of the ideological spectrum^§^: (liberals: 26.4%; t(477) = −11.46, *P* < 0.001, and 95% CI = [23.1%, 29.9%]; conservatives: 24.1%; t(235) = −8.59, *P* < 0.001, and 95% CI = [19.6%, 29.2%]). The results from the win-win condition were also consistent and significant across these robustness checks (all *P*s < 0.001). See Table 2 for a summary of results across all studies.

**Table 2.** Summary table for studies 1–5 of the percentage of participants preferring to support the opposing group (for lose-lose choices) and support the in-group (for win-win choices; study 1 only)

	Lose-Lose (% choosing to support opposing group)	Win-Win (% choosing to support in-group)	Participant attitudes
**Study 1**			
Abortion access	27.8%	81.9%	Pro-choice: 76%; Pro-life: 24%
Gun control	25.3%	68.3%	Anti-gun ownership: 75%; Pro-gun ownership: 25%
US politics	20.8%	67.6%	Democrats: 70%; Republicans: 30%
UK politics	32.7%	72.1%	Labor party: 64%; Conservative party: 36%
**Study 2** Abortion access	35.1%		Pro-choice: 74%; Pro-life: 26%
**Study 3** US politics	36.4%		Democrats: 65%; Republicans: 35%
**Study 4** US politics			
Control	41.1%		Democrats: 60%; Republicans: 40%
Identity-strengthened	30.4%		Democrats: 64%; Republicans: 36%
**Study 5** Abortion access			
Control	39.2%		Pro-choice: 67%; Pro-life: 33%
Norm-subtract	36.7%		Pro-choice: 71%; Pro-life: 29%
Norm-add	57.7%		Pro-choice: 72%; Pro-life: 28%

For study 2, the percentage captures the proportions of participants with an indifference amount of less than $1 (i.e., would rather add $1 to the opposing group than subtract $1 (or less) from the in-group).

Although individuals prefer to deduct funding from their in-group rather than support the opposing group in this study with real consequences, one possible explanation is that participants view this as the better outcome for their cause. Indeed, if individuals believe that the opposing group is more effective at advancing their interests per dollar spent than their own group, then reducing equivalent funding to the in-group maximizes the relative difference in outcomes between groups in a lose-lose decision. However, our results show the reverse: Participants indicated that they view organizations supporting their side of an issue to be *more* effective in spending donation money to achieve their goal (*SI Appendix*, Results for Study 1). To verify that our results are not driven primarily by individuals who believe the opposition is more effective with funds, we conducted an ancillary analysis in which we studied the subset of participants who believe organizations on their side of an issue are strictly more effective with funds (43.8% of observations). These participants in the lose-lose condition *still* preferred to “harm” their side (supporting the opposing side was chosen by only 22.8% of these participants; t(402) = −10.82, *P* < 0.001, and 95% CI = [19.2%, 27.0%]).

Finally, we conducted an initial test of our central theory that individuals make decisions in group conflicts on the basis of protecting their identity (developed in-depth in Studies 3 and 4). As previously outlined, individuals believe that acts of support are more value-expressive than acts of opposition. Individuals may choose the least value-expressive option when offered two unfavorable choices, thereby opposing their own group rather than supporting the opposing group. We predicted that this desire to protect their identity (and therefore the choice not to support the opposition) should be stronger for participants with stronger group identities, as assessed by stronger attitudes about the underlying issue. Indeed, in the lose-lose condition, we find that those with strong attitudes were even less likely to choose to add $1 to the opposing side (vs. subtract $1 from their side), compared to those with medium (β = 0.89, SE = 0.17, z = 5.21, *P* < 0.001, OR = 2.43, and 95% CI = [1.74, 3.40]) or weak (β = 1.05, SE = 0.18, z = 6.01, *P* < 0.001, OR = 2.87, and 95% CI = [2.04, 4.05]) attitudes. In the win-win condition, we find that those with strong attitudes were more likely to choose to add $1 to their own side (vs. subtract $1 from the opposing side), compared to those with medium (β = −0.36, SE = 0.17, z = −2.13, *P* = 0.03, OR = 0.70, and 95% CI = [0.50, 0.97]) or weak (β = −0.83, SE = 0.16, z = −5.10, *P* < 0.001, OR = 0.44, and 95% CI = [0.32, 0.60]) attitudes.

Taken together, these results establish that not only do group members prefer to deduct funds from their in-group rather than contribute an equivalent amount to their opposition, but they make this choice despite explicitly believing that this leaves their group worse off than the alternative. While motives such as harm minimization, in-group favoritism, and in-group love cannot explain the findings from this study, the results are consistent with predictions from the Identity-Support model. Having established that our findings in win-win scenarios are consistent with previous work, we focus on decision-making in our novel lose-lose paradigm in the remaining studies.

### Study 2: Quantifying the Aversion to Supporting an Opposing Group.

Although Study 1 establishes that individuals prefer to deduct a given amount of funds from their in-group rather than add the same amount to the opposition, Study 2 quantifies the degree to which individuals prefer to harm their own group rather than support an opposing group. We approach this quantitative analysis by eliciting participants’ indifference amount between harming their own group and supporting the opposition using an incentive-compatible choice titration procedure (34; see *Methods* section for more detail). For this choice titration analysis, we recruited 300 US participants from Amazon’s Mechanical Turk with a final sample of 268 following our pre-registered exclusions. Given that we found consistent evidence across issues in Study 1, here we focused on a single polarizing issue: abortion access.

We informed participants that the researchers would make two $10 donations: one to a pro-life organization and another to a pro-choice organization. Participants were asked to choose how to alter the donation amount in a series of 14 choices. For each choice, they could select to either add $1 to the opposing organization’s donation or subtract an amount (sequentially from $0.10 to $10, order counterbalanced) from their own side’s organization (similar to a price list; 35). To incentivize responses, we informed participants that for one in ten participants, chosen at random, we would actually make donations to both organizations and randomly select one of their 14 choices to alter the donation amount (for similar elicitation and bonus procedures, see refs. 36–38). As in Study 1, participants also reported their beliefs about the relative effectiveness of pro-life and pro-choice organizations, as well as their attitude strength toward the issue.

**Fig. 2.**
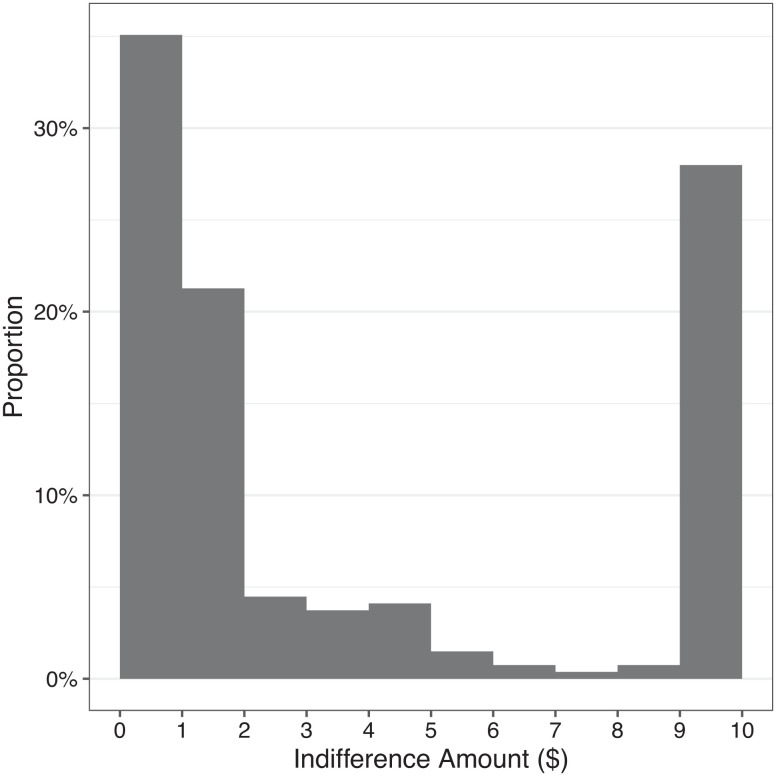
Results from Study 2 (N = 268): distribution of participants’ indifference amounts.

Our results show that participants’ indifference amounts had a mean value significantly greater than $1 (M = $3.85, median = $1.50, max = $10, SD = $4.10, t(267) = 11.39, *P* < 0.001, d = 0.70, and 95% CI = [0.56, 0.83]), and the majority of participants (65%) had an indifference amount greater than $1 (χ^2^(1, N = 268) = 23.29, *P* < 0.001; see Fig. 2). In other words, on average participants required almost $4 to be subtracted from the donation going to their organization, to be indifferent toward adding $1 to the opposing organization. Strikingly, 28% of all participants chose to entirely forgo the $10 donation to their side rather than add $1 to the opposing organization. Lastly, we found that those with greater attitude strength (i.e., more strongly pro-choice or pro-life) required more funds to be subtracted from their side to be indifferent toward adding $1 to the opposing side, and we also replicated the finding that participants believed that organization on their side is more effective at spending their donation money to achieve their goal (for details on both results, *SI Appendix*, Results for Study 2).

In sum, Study 2 demonstrates the strength of participants’ preferences when facing lose-lose choices. Despite believing that their own side is more effective with funds, group members preferred to subtract, on average, more than three times as much from their own group rather than give a small amount of support to the opposing group.

### Study 3: Identity Concerns Trump Effectiveness Considerations for Lose-Lose Decisions.

Studies 1–2 provide evidence across contexts that individuals are so averse to supporting an opposing group that they prefer to harm their own group instead. Moreover, the finding that individuals with stronger attitudes toward the focal issue (and therefore stronger identity-relevance; 39, 40) are more prone to harming their in-group rather than helping the opposing group offers preliminary evidence that identity considerations govern decision-making in group conflicts. In Study 3, we directly test the hypothesis that identity concerns, as opposed to effectiveness considerations, underlie the psychology of decision-making in group conflicts involving lose-lose choices.

To test the relative contributions of identity concerns and effectiveness considerations, we recruited 400 US participants from Amazon’s Mechanical Turk with a final sample of 393 following our pre-registered exclusions. After indicating whether they identified more strongly as Republican or Democrat (binary choice), participants were asked to make a lose-lose choice, identical to the US political party choice from Study 1. Subsequently, participants responded to an effectiveness and an identity concern question, both on a seven-point Likert scale, asked in a randomized order. The effectiveness question was similar to the one used in the previous studies, and the identity concern question asked participants whether adding $1 to the opposing side or subtracting $1 from their side “would make you feel like a worse [Democrat/Republican]? In other words, which option most undermines your identity as a [Democrat/Republican]?”.

As in our previous studies, less than half of participants chose to add $1 to the organization supporting the opposing political party (36.39%), χ^2^(1, N = 393) = 38.59, and *P* < 0.001, for both Democrats and Republicans (both Ps < 0.001). Moreover, as in the previous studies, the overall effectiveness measure was positive (M = 0.61, SD = 1.35), t(392) = 8.95, *P* < 0.001, d = 0.45, and 95% CI = [0.35, 0.55]), indicating that, on average, participants view the organization on their side to be more effective at using donated funds to pursue their mission.

**Fig. 3.**
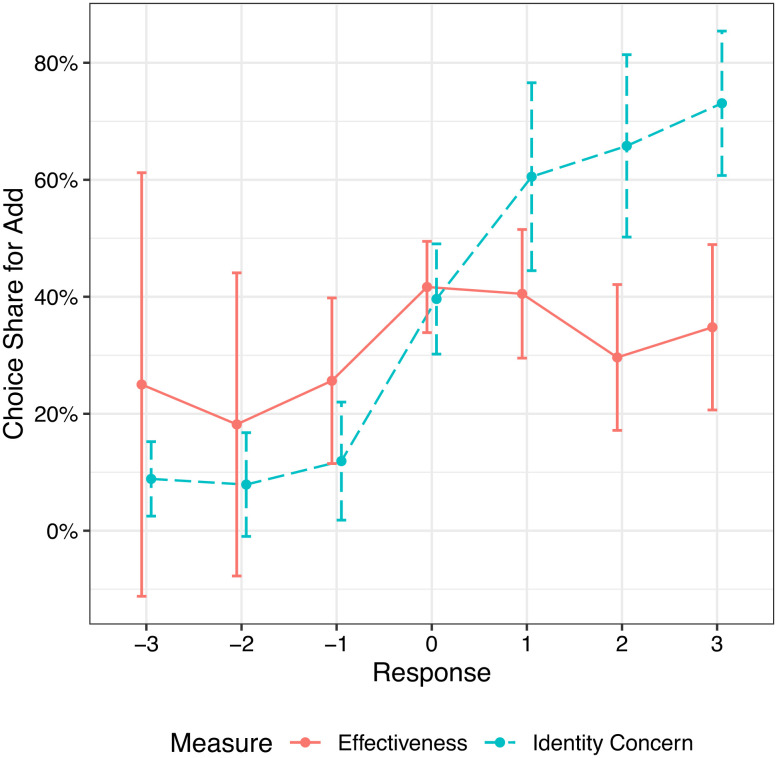
Results from Study 3 (N = 393): choice share by measure. The vertical axis shows the proportion of participants who chose to add funds to the donation for the opposing organization (vs. subtract from their side). The horizontal axis captures participants’ responses on the effectiveness and identity concern measures (which were both centered at 0). For the effectiveness measure, more positive values indicate the in-group is more effective at using donated funds (vs. the out-group; 0 indicates equal effectiveness). For the identity concern measure, more positive values indicate subtracting $1 from the in-group undermines identity more (vs. adding $1 to the opposing group; 0 indicates both equally undermine identity). Error bars represent 95% confidence intervals.

Critically, the identity concern measure revealed that participants believed adding $1 to the opposition undermined their partisan identity more than deducting $1 from their own party (M = −0.22, SD = 1.99, t(392) = −2.16, *P* = 0.03, d = −0.11, and 95% CI = [−0.21, −0.01]). The fact that individuals perceive their group-based identities to be at greater risk when supporting an opposing group (vs. harming their own) offers a clear rationale for why they choose in-group harm over supporting the opposing group. However, to explicitly test the contributions of identity concerns and effectiveness considerations on choice, we regressed participant choice (0 = subtract $1, 1 = add $1) on both our identity and effectiveness measures using a logistic regression. The identity concern measure was positively associated with choice, such that participants were more likely to choose to add to the opposing side when they believed that subtracting $1 from their side undermined their identity more (β = 0.65, SE = 0.07, z = 8.77, *P* < 0.001, OR = 1.91, and 95% CI = [1.66, 2.22]). The effectives measure was not significantly associated with choice (β = −0.09, SE = 0.09, z = −0.97, *P* = 0.33, OR = 0.91, and 95% CI = [0.76, 1.09]). A similar pattern of results was observed when choice was regressed on each measure in separate regressions. See *SI Appendix*, Results for Study 3 for regression results and Fig. 3 for plots of both measures.

In summary, Study 3 explicitly establishes that individuals feel that their group identity suffers more when supporting the opposition than when harming the in-group. Moreover, in a direct test of the relative contributions of identity concerns and effectiveness considerations, we find that whereas the identity measure is significantly correlated with the choice to harm the in-group, there is no significant association between the effectiveness measure and individual decision-making in lose-lose group conflicts.

These findings align with our Identity-Support model of group decision-making, pointing to the important role identity plays in decisions involving intergroup conflict. In a pre-registered supplemental study (*SI Appendix*, Study S1), we test another key element of the model, building on relevant research (18)—that acts of support are more value-expressive than acts of opposition. Participants read about and evaluated another participant from their in-group (Republican or Democrat) who had to make either a lose-lose or win-win allocation (as in the US political party choice from Study 1) and were told which option the participant chose. Based on this decision, they were asked to assess how strongly they believed this target identified with their political party. In the win-win scenario, those who opted to support their in-group were perceived as identifying more strongly with their party than those who subtracted money from the opposing group. In the lose-lose scenario, those who opted to support the opposing side were perceived as more weakly identifying with their party than those who subtracted from their in-group. This suggests that participants do in fact believe that acts of support are more value-expressive than acts of opposition using our paradigm. In line with the Identity-Support model, participants should prefer the choice that best promotes or protects their identity and therefore choose the most value-expressive option (supporting their own side) when offered pro-attitudinal choices and avoid the most value-expressive option (supporting the other side) when offered counter-attitudinal choices.

### Study 4: Increasing the Salience of a Group-Based Identity Decreases the Probability of Supporting the Opposing Group.

Although Study 3 offers correlational evidence that identity considerations govern the preference to harm one’s in-group to avoid supporting the opposition, in Study 4 we causally test the relationship between identity concerns and choice in lose-lose group conflicts. Building on previous work showing that identities are malleable and making certain identities more salient can affect preferences and behaviors (15, 41, 42), we hypothesized that strengthening group identity salience would lead to an increase in the probability of harming one’s own group to avoid supporting the opposing group.

To test our hypothesis, participants were randomized into one of two conditions: In the identity-strengthened condition, participants were asked to write about an event, story, or personal experience where they strongly identified with their political party. In the control condition, participants wrote about what they do on a typical Monday evening. Participants then made the same choice as in Study 3—add $1 to the donation going to the organization supporting the opposing political party or subtract $1 from the donation going to the organization supporting their own political party.

**Fig. 4.**
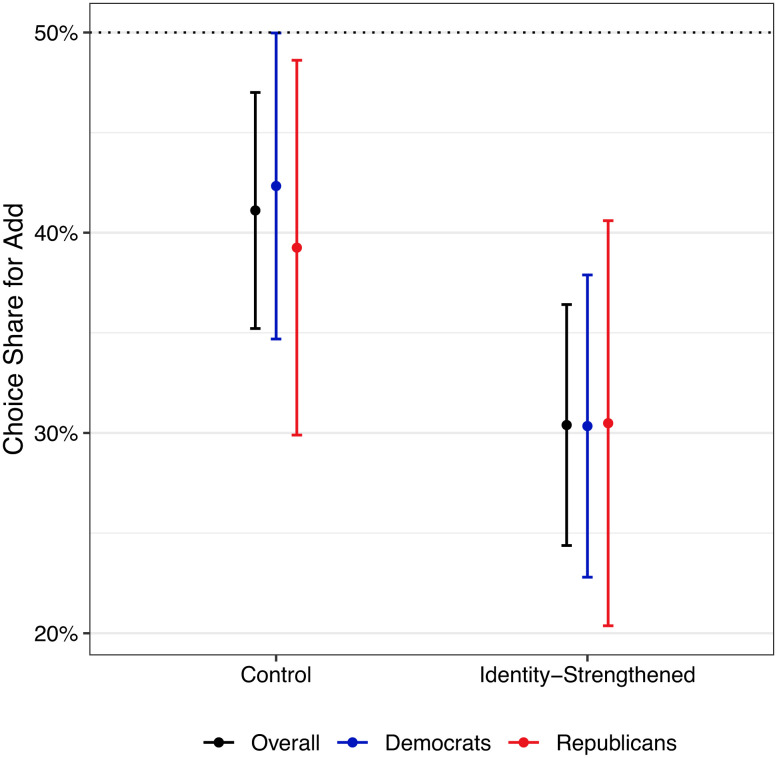
Results from Study 4 (N = 497): choice share by condition. The vertical axis shows the proportion of participants who chose to add funds to the donation to the opposing organization (vs. subtract from their side). Error bars represent 95% confidence intervals.

We recruited 500 participants from Amazon’s Mechanical Turk, with a final sample of 497 following our pre-registered exclusions. Consistent with our previous studies, in the control condition, the proportion of participants choosing to add $1 to the organization supporting the opposing political party was less than 50% (41.11%), χ^2^(1, N = 270) = 8.18, and *P* = 0.004. In the identity-strengthened condition, the proportion of participants choosing to add $1 was significantly lower (30.4%) than in the control group, χ^2^(1, N = 497) = 5.67, *P* = 0.017, and ϕ = 0.11 (see Fig. 4). This result held among the subset of participants who believed organizations on their side of a cause are more effective with funds (74% of participants; χ^2^(1, N = 369) = 5.36, *P* = 0.021, and ϕ = 0.13), Democratic participants (62% of participants; χ^2^(1, N = 308) = 4.24, *P* = 0.039, and ϕ = 0.12), and was directional but non-significant among the (relatively smaller) subset of Republican participants (38% of participants; χ^2^(1, N = 189) = 1.20, *P* = 0.274, and ϕ = 0.09). As in prior studies, we also found that participants believed that organizations on their side are more effective at spending their donation money to achieve their goal (*SI Appendix*, Results for Study 4). In sum, our results provide additional evidence for the role of identity on decision-making in group conflict, demonstrating a causal effect of identity salience on the decision to harm one’s in-group rather than support the opposition.

Finally, we note that Study 4 was designed to also address the potential confound that participants may disproportionately focus on the most negative ways in which the opposing group would use donated funds, but do not similarly consider the most positive ways in which the in-group would use donated funds (43). Such a difference could explain the strong aversion to supporting the opposing side that we find across lose-lose choices (although would not explain the win-win preference to help one’s own side rather than harm the opposition). Consequently, in this study, we also specified that all donations would go to “administrative costs (e.g., maintaining the organization’s website)” to hold constant the use of donations, ensuring that participants would imagine similar donation uses for each organization. We therefore conclude that the preference to harm one’s own group rather than support an opposing group is not explained by different imagined uses of the funds by the in-group and opposing group.

### Study 5: Modulating Group Norms Alters Decision-Making in Group Conflicts.

In Study 5, we test a practical method for shifting behavior in lose-lose group conflict, specifically testing whether shifting perceived in-group norms alters individual decision-making. Since our results suggest that individuals make choices to protect and promote their group identity, we hypothesized that decision-making will be sensitive to group norm information. In the absence of clear norm information, we consistently find that individuals avoid supporting the opposing group. In Study 5, we test whether providing participants with alternate group norm information (i.e., others in your in-group chose to support the opposing group) will increase the choice share supporting the opposing group over harming their own side, as the norm serves as a powerful guideline for making choices that maintain an identity consistent with the in-group.

To test whether modulating group norms alters decision-making, participants in Study 5 were asked to report their position on abortion access (“very much against abortion access” to “very much in favor of abortion access”) and were then randomized into one of three conditions: control, norm-add, or norm-subtract. Participants in all conditions chose between adding $1 to a donation going to an organization supporting the opposing side or subtracting $1 from a donation going to their side of the issue. In the norm-add condition, participants were also informed that in a previous study, 70% of participants who shared their views on abortion access chose to add to the opposing side rather than subtract from their own and that one of those participants had said the following: “I care way too much about my cause to take money away from it.” In the norm-subtract condition, participants were instead told that 70% of previous participants on their side of the cause had chosen to subtract from their in-group rather than add to the opposing group. The statement from the previous participant was changed to: “I dislike the other side way too much to give them money.” Finally, as in our prior studies, each participant indicated which of the two sides of the cause they believe is more effective at pursuing its mission. We recruited 653 participants from Amazon’s Mechanical Turk, with a final sample of 635 following our pre-registered exclusions.

**Fig. 5.**
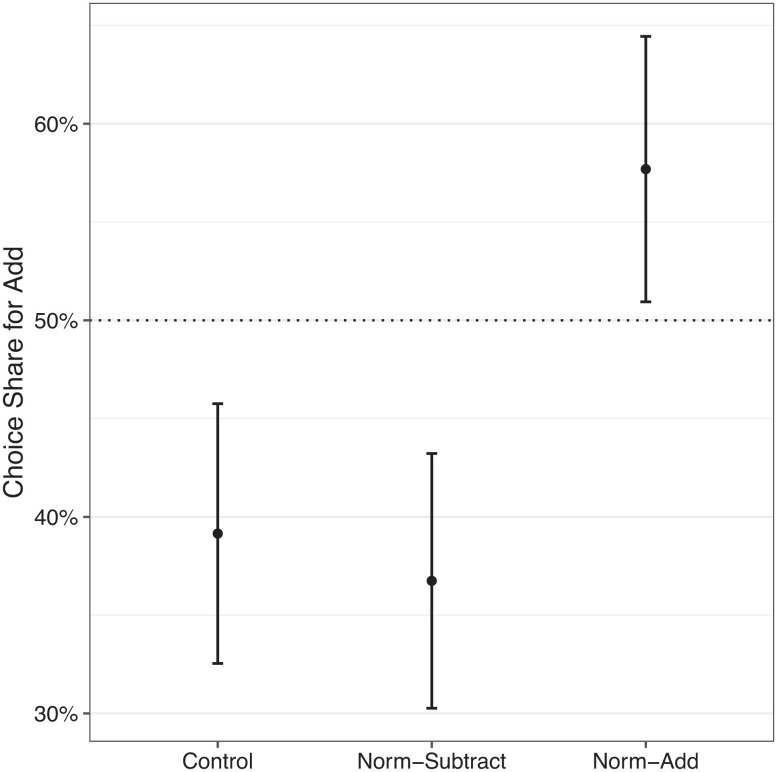
Results from Study 5 (N = 635): choice share by condition. The vertical axis shows the proportion of participants who chose to add funds to the donation to the opposing organization (vs. subtract from their side). Error bars represent 95% confidence intervals.

In the control condition, we replicated our fundamental finding: The proportion of participants choosing to add $1 to the opposing side’s donation was significantly less than 50% (39.2%), χ^2^(1, N = 212) = 9.55, and *P* = 0.002 (see Fig. 5).

In the norm-add condition, the key test of the power of group norms, we found a significant increase in the proportion of participants choosing to add $1 in the norm-add condition (57.7%), compared to participants in the control condition χ^2^(1, N = 420) = 13.72, *P* < 0.001, and ϕ = 0.19. In fact, the proportion of participants in the norm-add condition choosing to add $1 to the opposing side was significantly greater than 50% (χ^2^(1, N = 208) = 4.62, and *P* = 0.032). The results of the norm-add condition show that norms-based interventions about group identities can powerfully shift decision-making away from harming the in-group and toward supporting the opposing group.

In the norm-subtract condition, the proportion of participants choosing to add $1 to the opposing side (36.7%) was not statistically different from the control group, χ^2^(1, N = 427) = 0.17, *P* = 0.680, and ϕ = 0.02. The similarity between the norm-subtract and control conditions implies that in the absence of an explicit group norm (as in the control condition), the default norm is not to support the opposition even at the expense of harming one’s own side. We further verified that the norm-add and norm-subtract conditions were statistically different, χ^2^(1, N = 423) = 17.79, *P* < 0.001, and ϕ = 0.21. In a supplementary analysis (*SI Appendix*, Results for Study 5), we found that the norms manipulation can shift behavior even for those with strong attitudes toward an issue. We also replicated the result that participants believed the organization on their side is more effective at spending donation money to achieve their goal.

## Discussion

In the present work, we investigate how individuals prefer to adjudicate a lose-lose choice in intergroup conflict: harm their in-group or support their opposition. We operationalize this choice by giving study participants the option to either deduct funds from organizations within their in-group or add the same amount of funding to an opposing organization. Such choices help to separate various motives that could be driving decision-making, and remarkably, we find that even though individuals report that organizations in their in-group (vs. opposing group) are more effective with funds, they choose to deduct from their (more effective) in-group rather than add an equivalent amount of funds to the opposition. Indeed, individuals are so averse to providing any support to the opposing group that they, on average, accepted triple the amount of financial loss to their in-group to avoid any gains for the other side (Study 2). We reproduce our main findings across both sides of an array of group conflicts (abortion, gun control, political party) and in multiple countries (United States and United Kingdom; Study 1) to illustrate that the preference to harm one’s in-group to avoid supporting the opposing group is a robust, fundamental feature of individual decision-making in group conflicts.

Moreover, we explored the role of identity concerns to understand the psychology underlying the preference to harm one’s in-group rather than support the opposition. We found that whereas the strength of an individuals’ group identity strongly correlates with the decision to harm the in-group rather than support the opposition, individual assessments of group efficacy were uncorrelated with choice (Study 3). Manipulation of identity salience modulated the choice to harm the in-group vs. support the opposition (Study 4), further illustrating the central role of identity considerations in decision-making within group conflicts. Finally, we demonstrated a practical method to alter preferences in intergroup conflicts: Shifting perceptions of in-group norms lead to corresponding changes in behavior—individuals who were told that other in-group members were willing to support the opposing group became more likely to do the same (Study 5).

### Identity Concerns as the Central Driver of Decision-Making in Group Conflicts.

Previous models of individual psychology in groups, such as in-group favoritism (1, 3, 4, 44, 45) and in-group love (5, 6), examined decision-making using win-win scenarios, which cannot explain our findings in lose-lose scenarios. In a win-win context, in which individuals choose between various gains for the in-group and/or losses for the out-group, past work has found that individuals will seek the best relative outcome for their in-group (“in-group favoritism”) while avoiding unnecessarily harming the out-group (“in-group love” rather than “out-group hate”). While this literature used “minimal” groups where trivial differences created in-group and out-group distinctions, we replicate the preference to help one’s in-group rather than harm the out-group using natural groups for preexisting polarizing issues. However, we find that in a lose-lose context, individuals choose to financially harm their in-group rather than support an opposing out-group. This is a violation of both in-group love and in-group favoritism, as the alternative choice—supporting the opposition—maximizes the relative position of the in-group, because organizations on one’s side are generally viewed as more effective with funds than opposing organizations. In fact, participants even chose to accept triple the amount of financial losses to their own group to avoid supporting the opposition, illustrating that group members were not acting to establish the most favorable comparison between their in-group and the opposing group. Rather than in-group love, the results from lose-lose scenarios appear to be evidence for the opposite—out-group hate—in line with recent work on negative partisanship, finding that partisans are demonstrating increasingly negative affect toward the opposing party (28, 29). Among political partisans in the United States, “out-party hate” was recently found to be stronger than “in-party love” (30).

We synthesize prior work on support-framing (18, 19) and propose the Identity-Support model, which can parsimoniously explain our findings across win-win and lose-lose scenarios. The model suggests that individuals act in group conflicts to promote their identity, and they do so primarily by providing support to causes they believe in (and avoid supporting causes they oppose; see also *SI Appendix*, Study S1). Simply put, in win-win contexts, supporting the in-group is more expressive of one’s identity as a group member than harming the opposing group, thereby leading to a preference for in-group support. In lose-lose contexts, supporting the opposing group is more negatively expressive of one’s identity as a group member than harming the in-group, resulting in a preference for in-group harm. Therefore, the principle that individuals make decisions in group conflicts to promote and protect their identity, primarily by allocating their support in ways that most align with their values, offers a single framework that predicts individual behavior in group conflicts in both win-win and lose-lose contexts.

### Alternative Explanations and Related Literature.

Although our findings offer strong support for the role of identity considerations in group conflict, our results do not address whether these identity concerns are driven by a motivation to maintain or boost one’s self-image (46–48) or their reputation (i.e., for social approval; 49, 50). As an initial test of whether the aversion to supporting the opposing side is driven primarily by reputation concerns, we ran a pre-registered supplemental study (*SI Appendix*, Study S2). As in previous studies, we offered participants a lose-lose trade-off, but here we also manipulated whether the choice was explicitly anonymous or would be made public. If participants’ decision is based on public compliance or a desire for social approval (51, 52), we would expect the effect to be stronger when making their choice publicly (vs. privately). However, we found that individuals’ preferences did not differ when their choices were public vs. private and that they preferred to harm their group rather than help the opposing group in both conditions. Our results suggest that the motive to express one’s values by avoiding out-group support is internalized. However, some work suggests that social influence may still be at play as individuals sometimes act as though they are being observed by a third party even when they are not (as in the anonymous condition; 47, 53, 54, 55). Nevertheless, this supplemental study provides further evidence for the robustness of the aversion to helping the opposing side regardless of whether others would learn about their decision.

In the same supplemental study, we examined a possible alternative explanation for the pattern of preferences we observe. Participants in our experiments may have chosen to subtract funds from their side (rather than add to the opposing side) because it feels easier to undo or reverse (e.g., by making an additional donation to their side later). By contrast, participants may believe it is more difficult to “undo” the addition of funding to the opposing side. We therefore asked study participants to explain why they chose the option they selected. Of the 497 participants in the study, only four mentioned reversibility as an explanation for their choice to subtract funding, suggesting that this is not the primary driver for the preference to harm one’s own side in our studies.

This work studies decision-making for polarizing issues where individuals may have deeply held beliefs. We chose polarizing contexts because of the importance of improved decision-making around these contentious issues and our specific interest in intergroup conflict. While previous research finds that deeply held beliefs or sacred values lead to behavior that frequently departs from normative theory (56, 57), this prior work does not make clear predictions for which choice individuals will make in the lose-lose scenarios in our studies. That is, individuals with deeply held beliefs tying themselves to their in-group would likely have a strong aversion to both harming the in-group and helping the out-group, though it is unclear which would prevail based on this literature. We also note that the preference to harm one’s in-group persists even among individuals for whom these are not strongly held beliefs (i.e., those who report weaker attitudes toward their side of the issue). We would not expect the preference to harm one’s own group rather than help an out-group to emerge for all out-groups, but rather for out-groups with which individuals do not want to align themselves (58, 59) or for groups that directly oppose the decision-maker’s beliefs.

### Implications for Better Outcomes in Group Conflicts.

One striking facet of our work is that individuals resolve lose-lose decisions in group conflicts in ways that leave their own in-group in a worse relative position than if they had simply supported the opposition. When generalized across both sides of several issues, our work points to the possibility that identity concerns may act as a barrier to better outcomes for both groups. Therefore, groups engaged in conflict may realize mutual gains if individuals are less averse to supporting the opposition. Building on an extensive literature on in-group norms (60–63), we demonstrated that shifting group norms can modulate individuals’ aversion toward showing support for the opposing group. While many accounts suggest that the United States is becoming more affectively and ideologically polarized (64–66), an emerging literature on “false polarization” suggests that intergroup conflict is exacerbated by misperceptions about the magnitude and consistency of out-group members’ beliefs (67, 68). In fact, recent work finds that Americans often tolerate and even show admiration for in-group members who seek to understand the out-group (69), indicating both sides may have a desire for cooperation. Our findings offer a practical approach that has the potential to increase cooperation: Providing information about in-group norms may reduce group members’ identity concerns, thereby allowing for behaviors that support the out-group when advantageous. Future research might further examine the nature of this norm belief and test realistic and effective methods for increasing the likelihood to work with the opposition, such as modeling cooperative behavior by high-status in-group members.

Our findings add to a literature on how psychological barriers impede the advancement of important causes (13). In contexts in which accommodating two groups’ desires is crucial for progress, how do we compromise when both sides would rather harm their own cause than make concessions in which the opposition gains any benefits? For example, a congressperson wishing to cross the aisle to support legislation may be hindered by the assumption that it would be a sign of disloyalty to her constituents. In an era of high perceived polarization, understanding how identity concerns and beliefs about group norms shape these decisions is critical. Otherwise, these psychological barriers are likely to impede progress, not only for the causes we oppose, but also for those we most strongly support.

## Materials and Methods

### Overview.

All experiments were approved by the UC San Diego Institutional Review Board (IRB), and all participants gave their informed consent to participate.

### Studies 1A and 1B.

Study 1A was conducted in January 2022. As outlined in our pre-registration (https://aspredicted.org/cz62t.pdf), we aimed to recruit a nationally representative sample of 800 US participants through Prolific and ended up with a sample of 801 participants who completed the study (50.6% female, mean age = 45.17 y). We excluded four participants who failed the reading check, leaving us with a final sample of 797 participants.

Participants were randomized into one of two conditions (lose-lose or win-win) to make a hypothetical choice. All participants first reported their position on three issues, presented in a randomized order: abortion access (“very much against abortion access” to “very much in favor of abortion access”), gun control (“very much against gun control” to “very much in favor of gun control”), and political party (“strongly Republican” to “strongly Democratic”). Responses were captured using a six-point Likert scale to prevent participants from expressing indifference, as alignment to a side of each issue was required for the scenario assignment. We used these responses to classify participants as having either weak (3, 4 on the scale), medium (2, 5 on the scale), or strong (1, 6 on the scale) attitudes.

For each of the three issues, participants were told that, as part of the study, donations would be made to organizations supporting each side, and that they would need to make a choice about how to alter the donation amount. We informed participants that we would randomly select ten of them and adjust one of the donation amounts based on their choice and actually make the donations on their behalf. For each issue, brief descriptions of each organization’s mission were provided. For example, for the abortion access issue, participants read: “The mission of the Pro-life organization is to reduce access to abortions. The mission of the Pro-choice organization is to increase access to abortions.” No organizations were referred to by name to avoid any associations a participant may have with a particular organization. All the scenarios and corresponding binary choices were presented in a randomized order.

For each cause, participants were asked to select one of two options. In the win-win condition, both options altered the donation in ways that were favorable given the participant’s stated attitude: either add $1 to the donation going to the organization on their side or subtract $1 from the donation going to the organization on the opposing side. In the lose-lose condition, both options altered the donation in ways that were unfavorable given the participant’s stated attitude: either add $1 to the donation going to the organization on the opposing side or subtract $1 from the donation going to the organization on their side.

After responding to all three scenarios, participants reported which side of each issue had organizations that they believed to be more effective at pursuing their mission. Participants were specifically asked “which one is able to do more with each dollar they receive?”. Responses were collected on a seven-point Likert scale for all three issues, in a randomized order: abortion access (“pro-life organization are more effective” to “pro-choice organizations are more effective”), gun control (“pro-gun organizations are more effective” to “anti-gun organizations are more effective”), and political party (“Republicans are more effective” to “Democrats are more effective”).

Study 1B was conducted in February 2022 and was identical, except we collected participants from the UK, and only focused on a single issue—political partisanship. As outlined in our pre-registration (https://aspredicted.org/m4nq5.pdf), we recruited a nationally representative sample of 400 UK participants through Prolific (50.3% female, mean age = 44.68 y). We excluded seven participants who failed the reading check, leaving us with a final sample of 393 participants. All participants reported their political position on the following six-point Likert scale: “Strongly Conservative Party” to “Strongly Labor Party.” As with Study 1A, we used these responses to classify participants as having either weak (3, 4 on the scale), medium (2, 5 on the scale), or strong (1, 6 on the scale) attitudes.

For analyses across issues, we combined the datasets collected from Studies 1A and 1B. To test whether attitude strength moderated the participants’ choices, we regressed their choice (0 = subtract $1, 1 = add $1) on their condition and the interaction between condition and attitude strength (as a categorical variable), using a logistic regression.

### Study 2.

Study 2 was conducted in December 2020. We recruited 300 US participants from Amazon’s Mechanical Turk (MTurk; 53% female, mean age = 36.71 y). As outlined in our pre-registration (https://aspredicted.org/cn7ry.pdf), we excluded participants who switched more than once between the left and right-hand choices (10.7% of participants). This fraction of exclusion is within the typical range observed in prior studies involving multiple price lists (70). All remaining participants passed the pre-registered reading check, and there were no duplicated MTurk IDs, so there were no additional exclusions, resulting in a final sample of 268 participants which was used for all analyses.

**Fig. 6.**
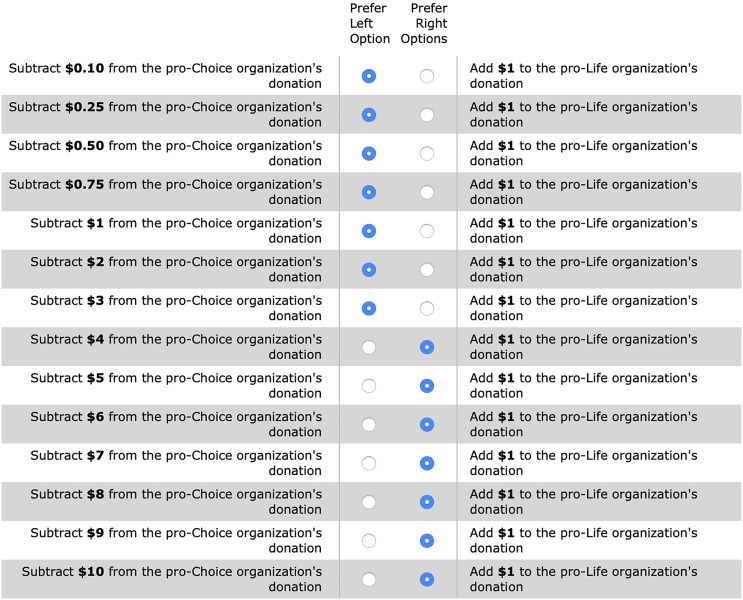
Screenshot of the series of choices made by participants in Study 2. This example is for a pro-choice participant, with the choices listed in ascending order.

All participants reported the extent to which they are against or in favor of abortion access on a 12-point Likert scale (“very much against abortion access” to “very much in favor of abortion access”). We informed participants that the researchers would make two $10 donations: one to a pro-life organization and another to a pro-choice organization. Participants were then asked to choose how to alter the donation amount in a series of 14 choices, where for each choice they could select either a right-hand side or left-hand side option (similar to a price list; 35). The right-hand side option was always to add $1 to the donation going to the opposing organization. The left-hand side option was to subtract $X from the donation going to the organization on the participant’s side of the cause, where X took the values 0.10, 0.25, 0.50, 0.75, 1, 2, 3, 4, 5, 6, 7, 8, 9, and 10 (see Fig. 6). We randomized whether participants viewed X in ascending or descending order. To incentivize the responses, we informed participants that for one in ten participants, chosen at random, we would actually make donations to both organizations and randomly select one of their 14 choices to alter the donation amount.

The outcome of interest was each participant’s indifference amount. We assumed that the indifference amount is at the midpoint of the subtract amounts on either side of the switch. For example (see Fig. 6), if a participant switches from preferring to subtract $3 from their side (instead of adding $1 to the other side), to preferring to add $1 to the other side (instead of subtracting $4 from their side), the indifference amount must be in the interval between $3 and $4 and was coded as the midpoint ($3.50). However, our results are robust to coding the indifference amount as the lower bound of each interval instead of the midpoint. Using the lower bound is a highly conservative measure since it might underestimate each participant’s true indifference amount, which lies in between the end points of each interval. If a participant selected the left-hand option for every choice, we coded their indifference amount as $10. If a participant selected the right-hand option for every choice, we interpreted their indifference amount to be between $0 and $0.10 and coded their indifference amount as the midpoint ($0.05). There was no significant difference in indifference amounts by price list order (*P* = 0.40), so we collapsed across the ascending and descending conditions.

After this series of choices, we assessed beliefs about the relative effectiveness of the organizations as in Study 1. Participants were asked “Do you believe that the Pro-Life or Pro-Choice organization is more effective at pursuing their mission? In other words, which one spends their donation money more effectively?”. Responses were collected on a seven-point Likert scale, allowing participants to report equal effectiveness.

### Study 3.

Study 3 was conducted in February 2022. As outlined in our pre-registration (https://aspredicted.org/ja6un.pdf), we aimed to recruit 400 U.S. MTurk participants and ended up with a sample of 401 participants who completed the study (51% female, mean age = 39.57 y). We excluded 2.0% of participants who failed the reading check or had duplicate MTurk IDs, leaving us with a final sample of 393 participants.

Participants first indicated whether they identified more strongly as Republican or Democrat (binary choice) and were subsequently asked to make a lose-lose choice, identical to the US political party choice from Study 1. We told participants we would pick ten of them at random and make the donations according to their adjusted donation amounts. Subsequently, participants responded to an effectiveness question and an identity concern question, in a randomized order. The effectiveness questions asked participants, “Do you believe that the Republican or Democratic Party is more effective at using donated funds to pursue their mission? In other words, which one is able to do more with each dollar they receive?” (1 = Republicans are more effective, 7 = Democrats are more effective). The identity question asked, “Which of these two options would make you feel like a worse [Democrat/Republican]? In other words, which option most undermines your identity as a [Democrat/Republican]?” (1 = Definitely adding $1 to the [Republican/Democratic] organization, 4 = Both choices equally undermine my identity as a [Democrat/Republican], 7 = Definitely subtracting $1 from the [Democratic/Republican] organization). The first option in square brackets was selected for participants who identified as Democrats, and the second was selected for Republicans.

### Study 4.

Study 4 was conducted in June 2021. As outlined in our pre-registration (https://aspredicted.org/gu45s.pdf), we recruited 500 U.S. MTurk participants (50% female, mean age = 39.51 y). We excluded 0.6% of participants who failed the reading check or had duplicate MTurk IDs, leaving us with a final sample of 497 participants. All participants reported whether they identified more strongly as Republican or Democrat (binary choice). Participants were then randomized into one of two conditions: control or identity-strengthened. Participants in the identity-strengthened condition were then asked to write about an event, story, or personal experience where they strongly identified with their political party. In the control condition, participants were asked to write about what they do on a typical Monday evening.

Subsequently, all participants were asked to make a lose-lose choice, similar to Study 1—participants had to choose between adding $1 to the donation going to the organization supporting the opposing political party or subtracting $1 from the donation going to the organization supporting their political party. We also specified that all donations would go to administrative costs (e.g., maintaining the organization's website).

As with our previous studies, we also asked participants whether they believe Republican or Democratic organizations are more effective at pursuing their mission, using the same scale as Studies 1 and 2, except with six points.

### Study 5.

Study 5 was conducted in March 2020. As outlined in our pre-registration (https://aspredicted.org/cz2kf.pdf), we aimed to recruit 650 U.S. MTurk participants and ended up with a sample of 653 participants who completed the study (55% female, mean age = 36.18 y). We excluded 2.8% of participants who failed the reading check or had duplicate MTurk IDs, leaving us with a final sample of 635 participants.

All participants reported the extent to which they are against or in favor of abortion access on a 12-point Likert scale (“very much against abortion access” to “very much in favor of abortion access”). Participants were then randomized into one of three conditions: control, norm-add, or norm-subtract.

In the control condition, participants were informed that the experimenter would be making donations to a pro-life and a pro-choice organization, and that they would have to choose how to alter the amount—add $1 to the donation going to the opposing side or subtract $1 from the donation going to their side. The norm-add condition was identical, except we also informed participants that in a previous study, 70% of MTurkers who shared their views on abortion access chose to add to the opposing side rather than subtract from their own and that one of those participants had said the following: “I care way too much about my cause to take money away from it.” In the norm-subtract condition, participants were instead told that 70% of previous participants on their side of the cause had chosen to subtract from their in-group rather than add to the opposing group. The statement from the previous participant was changed to: “I dislike the other side way too much to give them money.”

As with our previous studies, we also asked participants to indicate which of the two sides of the cause they believe is more effective at pursuing its mission, using a six-point scale.

## Supplementary Material

Appendix 01 (PDF)

## Data Availability

All data, analysis code, research materials, and pre-registrations have been deposited in Open Science Framework (Center for OpenScience) (https://osf.io/gzxke/?view_only=ea4d7c32e4b3499487d1c661fd4b5493).

## Footnotes

^*^While the term “lose-lose” is often used to express a loss for each side, here we define lose-lose (win-win) to describe situations where both options are unfavorable (favorable) to the decision-maker.

^†^We note that for this and all subsequent studies, we asked all participants to indicate their position on each issue without allowing them to express indifference. While this introduces noise by forcing truly indifferent participants to choose a side, it would not bias the results in a particular direction and works against finding an overall effect.

^‡^Statistical tests are from logistic regressions and account for repeated observations from each participant across issues by clustering the standard errors by participant.

^§^69% of participants in the US sample held either liberal (pro-choice, anti-gun ownership, and Democrat) or conservative (pro-life, pro-gun ownership, and Republican) positions on all three issues.
