# Supplementary material for: Individuals prefer to harm their own group rather than help an opposing group
Source: Proc Natl Acad Sci U S A. 2022 Nov 29;119(49):e2215633119. doi: 10.1073/pnas.2215633119 (PMC9894232; doi:10.1073/pnas.2215633119)
Supplement: Supplementary file 1 — Appendix 01 (PDF) [file pnas.2215633119.sapp.pdf]

PNAS

Supporting Information for

Individuals Prefer to Harm their Own Group rather than Help an Opposing Group.

Rachel Gershon and Ariel Fridman

Corresponding author: Rachel Gershon
Email: rgershon@ucsd.edu

This PDF file includes:

Supplemental Results for Study 1
Supplemental Results for Study 2
Supplemental Results for Study 3
Supplemental Results for Study 4
Supplemental Results for Study 5
Supplemental Study S1
Supplemental Study S2
SI References

Supplemental Results for Study 1

To test whether participants believed that organizations supporting their side of an issue are more effective at spending donation money to achieve their goal, we centered and re-oriented the effectiveness scale, such that 0 indicates equal effectiveness, positive values indicate organizations on the participant's side are more effective, and negative values indicate organizations on the opposing side are more effective. The overall effectiveness rating was positive ($M = 0.35$, $SD = 1.58$), $t(1,189) = 9.52$, $p < .001$, 95% CI = [.28, .43].

The belief that organizations within one's in-group are more effective with funds than those on the opposing side is consistent across each of the four issues and for participants on each side of the four issues ($Ps < .001$; Figure S1), however there are two notable exceptions. First, for pro-life Americans, the result does not attain statistical significance ($p = .43$) but is directionally consistent. The other exception is among "pro gun control" Americans, who indicate that they view organizations that are "anti gun control" as more effective ($M = -0.40$, $SD = 1.64$), $t(593) = -5.95$, $p < .001$, $d = -.24$, 95% CI = [-.33, -.16], such that individuals in this group are technically making a harm minimizing choice by not supporting the opposition. This deviation by "pro gun control" participants likely reflects the strong influence of the National Rifle Association and can serve as evidence that participant responses are not simply a result of motivated beliefs or partisan cheerleading (1), as they do appear to consider the real-world impact of such organizations, even when it is not in their favor. Despite these two exceptions, the overall pattern of results makes it clear that individuals generally perceive organizations on their side to be more effective than those on the opposing side.

Figure S1.

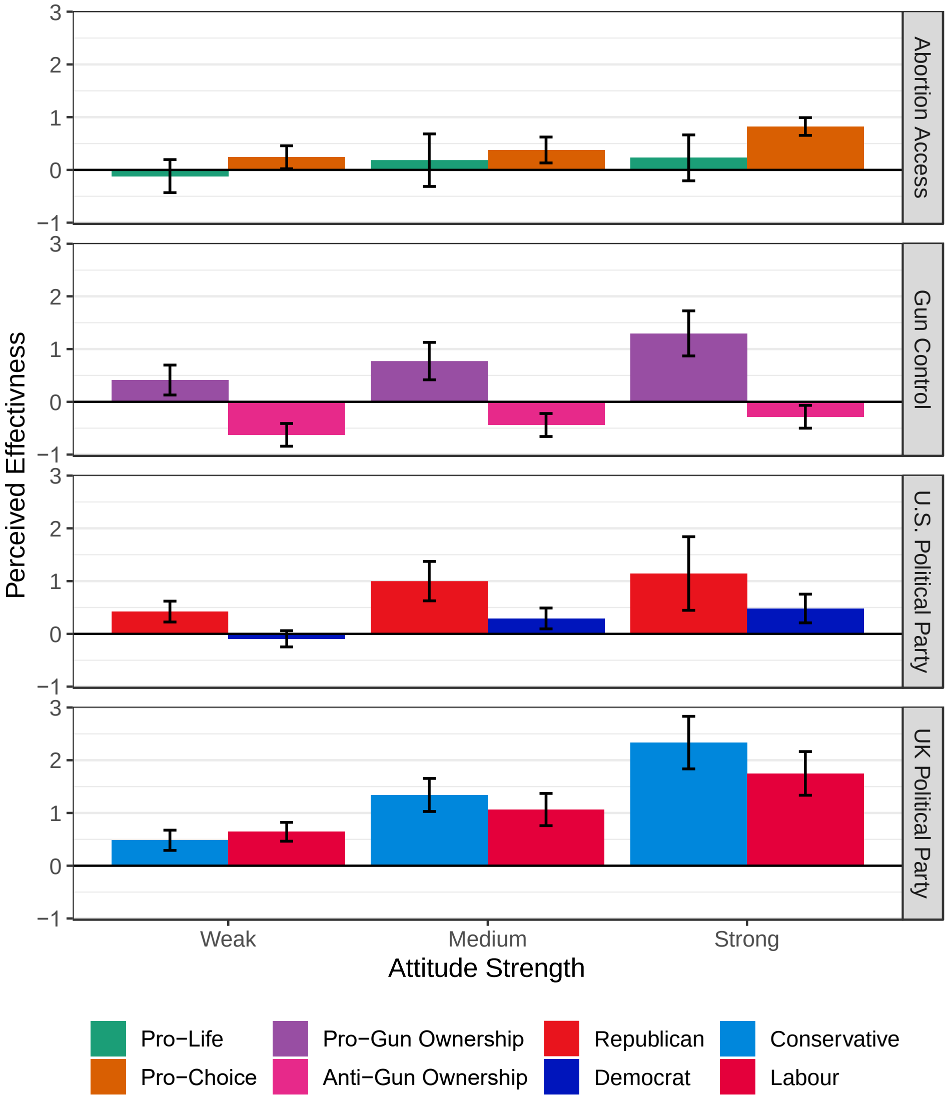

Fig. S1. Effectiveness measure from Studies 1A and 1B for all four issues by attitude strength. Positive (negative) values indicate that organizations on the participant's (opposing) side of a cause are more effective at managing their funds. Error bars represent 95% confidence intervals.

Supplemental Results for Study 2

We tested whether attitude strength moderates the reported indifference amounts. As in Study 1, we first centered and re-oriented the 12-point attitude strength scale, such that the middle numbers (6, 7) indicate the weakest attitude strength and values closer to the extremes indicate stronger attitudes (i.e., more strongly pro-choice or pro-life). In a linear regression model, we regressed participants' indifference amount on this adjusted attitude strength measure (as a continuous variable[1]). We found that greater attitude strengths were positively associated with indifference amounts ($\beta = .50$, $SE = .15$, $t(266) = 3.33$, $p < .001$, 95% CI = [.20, .79]; see Figure

[1] While we coded attitude strength as continuous, similar results were obtained when we created three buckets (weak, moderate, strong) and coded it as categorical as in Study 1, with the difference between weak and strong attitudes attaining statistical significance ($p < .001$).

S2). The correlation between attitude strength and indifference amount also addresses concerns of information leakage in the stimuli (2), wherein participants may make inferences from the fact that there were fewer choices less than (vs. greater than) $1 in the choice list. While prior work finds that those with weaker attitudes tend to be more susceptible to contextual effects (3), we find here that those with weaker (vs. stronger) attitudes subtracted less from their own group.
Figure S2.

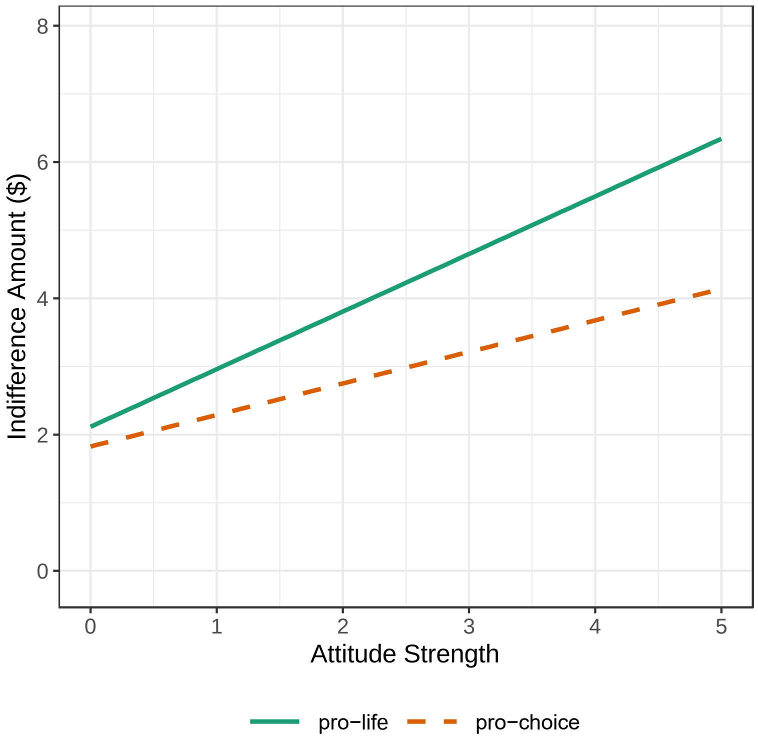

Fig. S2. Lines depict linear regression of indifference amount on attitude strength. Shaded regions represent 95% confidence intervals.

As in Study 1, we also tested whether participants believed that organizations supporting their side of an issue are more effective at spending donation money to achieve their goal. We replicated the result from Study 1, finding a positive overall effectiveness rating (M = 1.26, SD = 1.65), t(267) = 12.49, p < .001, d = .76, 95% CI = [.63, .90]). This pattern held significantly across pro-choice and pro-life participants (both Ps < .03). Indeed, most participants (64%) indicated that organizations on their side are more effective in spending their funds (χ^2(1, N = 268) = 20.99, p < .001), and 23% indicated equal effectiveness.

Supplemental Results for Study 3

See Table S1 for regression results for Study 3.

Table S1.

	Dependent variable: choice to add $1		
	(1)	(2)	(3)
intercept	-0.556***	-0.607***	-0.576***
	(0.133)	(0.123)	(0.116)
identity concern measure	0.647***	0.637***	
	(0.074)	(0.073)	
effectiveness measure	-0.09		0.028
	(0.092)		(0.078)
Observations	393	393	393
Log Likelihood	-204.154	-204.629	-257.589
Akaike Inf. Crit.	414.308	413.258	519.179
Note:	*p<0.05; **p<0.01; ***p<0.001		

Supplemental Results for Study 4

As in prior studies, we verified that the overall effectiveness rating was positive (M = 0.64, SD = 1.27), t(496) = 11.28, p < .001, d = .51, 95% CI = [.41, .60], indicating that participants believe that organizations supporting their political party are more effective than those on the opposing side. This pattern held across Democratic and Republican participants, and across both conditions (all Ps < .001).

Supplemental Results for Study 5

We investigated the efficacy of group norms for shifting decision making as a function of reported attitude strength, which was measured prior to the norms intervention. We conducted this analysis using a logistic regression, where we regressed participants' choice (0 = subtract $1, 1 = add $1) on the participants' condition (control, norm-add, norm-subtract), attitude strength (as a continuous variable), and their interaction. The results showed no significant interaction between condition and attitude strength (Ps > 0.40). However, there was a main effect of attitude strength, such that those with stronger attitudes toward the cause were more likely to harm their side than support the opposing side (β = -.28, SE = .08, z = -3.33, p < .001, OR = .76, 95% CI = [.64, .89]; see Figure S3). Nevertheless, these results imply that norms have the potential to shift behavior even for those with strong attitudes toward an issue.

Figure S3.

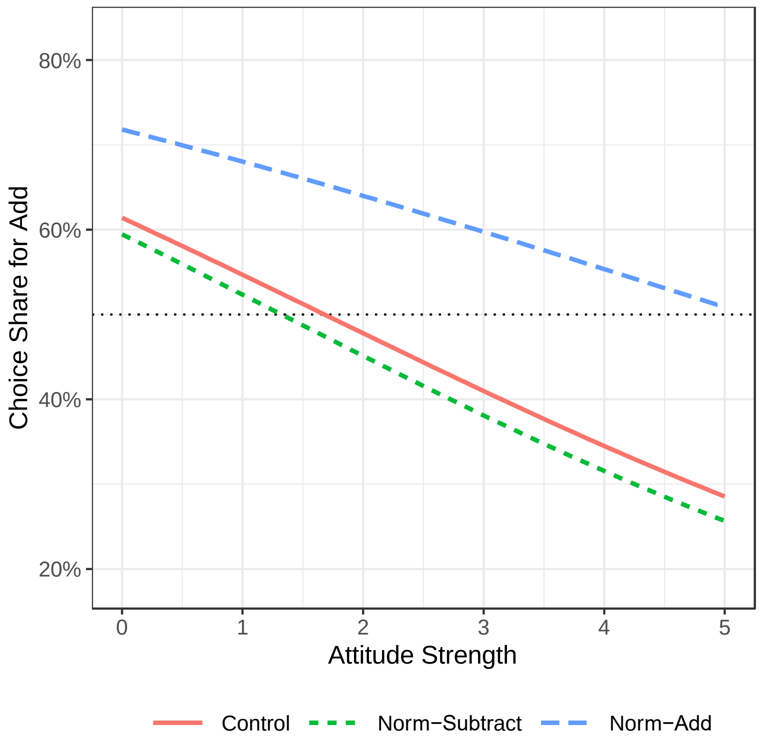

Fig. S3. Lines depict logistic regression of choice share on attitude strength by condition. Shaded regions represent 95% confidence intervals.

As a robustness check, we verified that all of our choice results replicated when limiting the sample to the 74% of participants who believed that organizations on their side of a cause are more effective with funds, and the only difference was that the proportion of participants in the norm-add condition choosing to add $1 was not statistically different from 50% at conventional levels (57.6%, $\chi^2(1, N = 165) = 3.06$, $p = .080$). Moreover, as in the previous studies, participants indicated that they viewed organizations supporting their side of a cause to be more effective in spending their donation money to achieve their goals. The overall effectiveness rating was positive ($M = 0.82$, $SD = 1.40$), $t(634) = 14.83$, $p < .001$, $d = .59$, 95% CI = [.50, .67], indicating that participants believe organizations on their side are more effective than those on the opposing side. This pattern held across pro-choice and pro-life participants, and across all three conditions (all Ps < .001).

Supplemental Study S1

The study was designed to test how people perceive others who have made either a win-win or lose-lose decision. Specifically, we tested whether evaluations of their identity as an in-group member differed based on how they chose, to shed light on whether people typically choose in ways that best protect others' perception of their identity.

Methods

Study S1 was conducted in August 2022. As outlined in our pre-registration (https://aspredicted.org/3wz2v.pdf), we recruited and obtained a sample of 400 U.S. MTurk participants who completed the experiment (49.5% female, mean age = 39.66 years). We

excluded 1% of participants who failed the reading check or had duplicate MTurk IDs, leaving us with a final sample of 396 participants.

The study had a 2 (choice type: win-win vs. lose-lose) x 2 (decision: add vs. subtract) between-subjects design. All participants first reported whether they identified more strongly as Republican or Democrat (binary measure). Next, in the win-win (lose-lose) condition, participants were told about another participant in a previous study who shared their political preference and faced a win-win (lose-lose) choice, and were informed about how they chose. In the win-win condition, the choice described was between adding $5 to a donation to support the in-group (which always matched the participant's political preference) or subtract $5 from a donation supporting the opposing group. In the lose-lose condition, the choices were reversed: add $5 to a donation to support the opposing group or subtract $5 from a donation supporting the in-group. Participants were also informed that this choice was incentivized.

Subsequently, all participants responded to the same question that formed the key dependent variable. Participants reported how strongly they thought the participant they were evaluating identified with their in-group (Democrat/Republican party) on a 7-point Likert scale (1 = identifies very weakly; 7 = identifies very strongly).

Results

As pre-registered, we regressed the dependent variable on condition (choice type and decision), and their interaction. We found that all regressors were statistically significant, and revealed the same pattern for Democrat and Republican participants. In the lose-lose condition, participants thought that those who had chosen to subtract $5 from their side identified more strongly with their in-group compared to those who added $5 to the opposing side ($\beta = 1.02$, $SE = .24$, $t(392) = 4.19$, $p < .001$). In the win-win condition, participants thought that those who had chosen to subtract $5 from the opposing side identified less strongly with their in-group than those who added $5 to their in-group ($\beta = -1.95$, $SE = .24$, $t(392) = -8.05$, $p < .001$).

Discussion

This study provides support for the identity-support model by demonstrating that actions of support are perceived as more powerful expressions of one's identity as an in-group member than actions of opposition. When faced with win-win choices, those who opted to support their side (vs. harm the opposing side) were perceived to be stronger in-group members. Similarly, when faced with a lose-lose choice (where both options are counter-attitudinal), the action of support to the opposing side (vs. harming the in-group) was seen as a stronger expression against the in-group.

In addition, consistent with findings from Study 1 that most participants prefer to harm their side when faced with lose-lose choices and help their side when faced with win-win choices, this study suggests that people's choices are consistent with those that best protect their identity as an in-group member, highlighting the role of identity in these decisions.

Supplemental Study S2

The goal of this study was to examine potential moderators of our main finding. Specifically, we tested whether varying the degree of anonymity of the choice (i.e., whether it is made in private or public) and the size of the stakes ($1 or $100) influenced choices.

Additionally, we asked participants to describe how they made their choice. In an exploratory analysis, we examined these free-responses to check for a possible alternative explanation for the pattern of preferences we observed: participants may have chosen to subtract

donations because it was the easier option to undo or reverse (e.g., by making an additional donation later).

Methods

Study S2 was conducted in January 2022. As outlined in our pre-registration (https://aspredicted.org/tj6qc.pdf), we recruited 500 U.S. MTurk participants and ended up with a final sample of 501 participants who completed the experiment (47% female, mean age = 39.79 years). We excluded 0.8% of participants who failed the reading check or had duplicate MTurk IDs, leaving us with a final sample of 497 participants.

The study had a 2 (anonymity: public vs. private) × 2 (stakes: $1, $100) between-subjects design. All participants reported whether they identified more strongly as Republican or Democrat on a 6-point scale ("strongly Republican" to "strongly Democratic").

In all conditions, participants were informed that the experimenter would be making a donation to a Democratic and Republican organization, and that they would have to choose how to alter the amount. In the public condition, participants were asked to imagine that their decision was not anonymous and that their friends would be informed of what they chose. In the private condition, participants were asked to imagine that the decision would be completely anonymous and nobody would ever find out what they chose. Next, all participants were asked to make a lose-lose choice, similar to Study 1. In the $1 condition, participants chose between adding $1 to the donation going to the opposing political party or subtracting $1 from the donation going their political party. In the $100 condition, the add and subtract amounts were both increased from $1 to $100.

Afterwards, all participants were asked to write a few sentences explaining why they chose the option they selected. Lastly, we asked participants whether they believe Republican or Democratic organizations are more effective at pursuing their mission, using the same scale as Study 1.

Results

Across all conditions, the proportion of participants who chose to add money to the opposing side was 38.0%, which was significantly different from 50%, $\chi^2(1, N = 497) = 28.02$, $p < .001$. There was no significant difference between Democratic or Republican participants ($p = .85$).

Anonymity. The proportion of participants who chose to support the opposing side in the public condition (35.5%) and private condition (40.1%) was not statistically different, $\chi^2(1, N = 497) = 1.15$, $p = .28$, $\varphi = .05$.

Stakes. The proportion of participants who chose to support the opposing side in the $1 condition (33.5%) was lower than in the $100 condition (42.9%; $\chi^2(1, N = 497) = 4.31$, $p = .04$, $\varphi = .10$.

To test for interactions between conditions, we regressed participants' choice on anonymity condition, stakes condition, and their interaction, using a logistic regression model. No significant interaction was found ($p = .71$).

Reversibility. The free responses were examined to determine whether participants subtracted funds from their side because they considered this action to be easier to reverse (e.g., through extra-experimental donations or fundraising actions) than adding to the opposing side. Four responses, amounting to less than 1% of participants, alluded to this motive in their explanation for their decision[2]. An additional participant, who chose to add funds to the opposing

[2] The 4 responses were: "… I felt like I could always donate money myself later to make up for the subtraction, whereas the reverse would not be possible. That made the choice to subtract more preferable."; "I could always donate another

side, also mentioned they would “make up for” their choice by adding funds to their side too at a later time[3], demonstrating that extra-experimental donations could be used to justify both adding and subtracting funds.

Effectiveness measure. As in the previous studies, participants indicated that they viewed organizations on their political side to be more effective at spending their donation money to achieve their goals. The overall effectiveness rating was positive (M = 0.71, SD = 1.43), t(496) = 11.13, p < .001, d = .50, 95% CI = [.41, .59], indicating that participants believe organizations supporting their political party are more effective than those on the opposing side. This pattern held across Democratic and Republican participants, and across all conditions (all Ps < .001).

Discussion

This study found that participants’ preference to harm their own group over supporting the opposing group is not influenced by degree of anonymity, suggesting that the motive to express ones’ values is internalized. We also found that the effect was attenuated with higher stakes ($100 vs. $1), though was not eliminated. This may suggest that harm minimization motives play a larger role when consequences are larger. We also did not find strong evidence that participants opted to subtract funds because it was more easily reversible than adding funding to the opposing side.

SI References

1. J. G. Bullock, A. S. Gerber, S. J. Hill, G. A. Huber (2013) Partisan bias in factual beliefs about politics. (National Bureau of Economic Research).

2. D. Prelec, B. Wernerfelt, F. Zettelmeyer, The Role of Inference in Context Effects: Inferring What You Want from What Is Available. *Journal of Consumer Research* **24**, 118-125 (1997).

3. H. Lavine, J. W. Huff, S. H. Wagner, D. Sweeney, The moderating influence of attitude strength on the susceptibility to context effects in attitude surveys. *Journal of Personality and Social Psychology* **75**, 359 (1998).

---

dollar to the republicans later to even it back out. I would never be able to have an opportunity to take away the dollar that I gave to the Democrats, though.”; “I would take away from my own party because I could help the party find new ways to gain ground and make up for the loss. Giving to the other party would simply give them more resources.”; and “…After withdrawing $100 from the Democratic Party, I plan to donate $200 to the Democratic Party by adding my donation.”.

[3] The response stated: “…I would be able to donate additional money to the Democratic organization at a later time to make up for this prior action.”
